# Obesity in short stem total hip arthroplasty using a minimally invasive supine anterolateral approach—a risk factor for short-term complications?

**DOI:** 10.1007/s00264-021-05079-1

**Published:** 2021-06-30

**Authors:** Matthias Luger, Rainer Hochgatterer, Clemens Schopper, Lorenz Pisecky, Jakob Allerstorfer, Antonio Klasan, Tobias Gotterbarm, Bernhard Schauer

**Affiliations:** Department for Orthopedics and Traumatology, Kepler University Hospital GmbH, Johannes Kepler University Linz, Krankenhausstrasse 9, 4020 Linz and Altenberger Strasse 69, 4040 Linz, Austria

**Keywords:** Short stem, Total hip arthroplasty, Minimally invasive, Anterolateral approach, Short-term complications, Obesity

## Abstract

**Purpose:**

Obesity is associated with increased risk for surgical complications in total hip arthroplasty (THA). The impact of obesity on short-term complication in minimally invasive (MIS) anterolateral approach is not well known. Therefore, this study was conducted to evaluate the early complications within the first 90 days after THA using a MIS anterolateral approach with a short-curved stem stratified by Body Mass Index (BMI).

**Patients and methods:**

A single centre consecutive series of 1052 hips in 982 patients (index surgery 2014–2019) with a short-curved stem and press fit cup implanted using a MIS anterolateral approach in supine position were screened for inclusion. Inclusion criteria were defined as end-stage primary osteoarthritis of the hip. Eventually, 878 implantations in 808 patients were included and stratified by body mass index (BMI). Peri-operative complications, within the first 90 days after surgery, were retrospectively evaluated.

**Results:**

Severely obese patients (BMI ≥ 35 kg/m^2^) and morbidly obese patients (BMI ≥ 40 kg/m^2^) demonstrated a significantly increased operation time (p < 0.001) and a higher risk for general surgical complications (p = 0.015) (odds ratio (OR) = 4.365; OR = 4.985), periprosthetic joint infection (PJI) (p = 0.001) (OR = 21.687; OR = 57.653), and revision (OR = 8.793; OR = 20.708).

**Conclusion:**

The risk for early PJI and overall surgical complications in MIS anterolateral approach is significantly increased in severely and morbidly obese patients. This leads to a significantly higher risk for revision surgery after index surgery within the first 90 days. A BMI above 35 kg/m^2^ is the clear threshold for increased risk of PJI in MIS anterolateral THA with a short curved stem. As the surgical complications are comparable to other approaches, MIS anterolateral short stem THA is also feasible with increasing BMI.

## Introduction

The incidence of obesity has grown in the western world in recent decades [1, 2]. The number of people with the highest body mass index (BMI) is increasing in size at the fastest rate, as evidenced by an annual increase of 50% in prevalence of patients with a BMI ≥ 40 kg/m^2^ [1, 3, 4]. Studies have shown the higher incidence of peri-operative complications in obese patients and higher rates of revision surgery in total joint arthroplasties in this patient cohort [5–7]. However, obese patients benefit significantly from total joint arthroplasty showing comparable clinical outcome scores following total knee arthroplasty compared to normal-weight patients [8, 9].

Minimally invasive approaches in total hip arthroplasty (THA) have gained more popularity over the last years allowing faster and less painful recovery with fewer post-operative precautions [10–12]. With the use of less invasive approaches, short hip stems are extensively used partly because of allowing tissue-sparing implantation [13, 14]. Apart from DAA, minimally invasive (MIS) anterolateral approach is established as a MIS approach in THA [15]. Compared to DAA, data on the peri-operative short-term complications in MIS anterolateral approach in obese patients are rare. Data suggests that the MIS anterolateral approach in THA might be responsible for an increased risk of periprosthetic fractures especially of the greater trochanter [16–18]. However, there is no consecutive case series on short-term complications in minimally invasive supine anterolateral approach with a short-curved stem stratified by BMI.

Therefore, the purpose of this study was to evaluate the short-term complications within 90 days after THA via a minimally invasive supine anterolateral approach with a short-curved stem stratified by BMI.

## Methods

### Patients

A retrospective evaluation of consecutive THAs at a single centre performed via a minimally invasive anterolateral approach using a cementless, curved short stem (Fitmore® stem, ZimmerBiomet, Warsaw, IN, USA) and cementless titanium press-fit cup with or without screws (Allofit®/-S, ZimmerBiomet, Warsaw, IN, USA) was carried out. The study was approved by the institutional review board (EK-No.: 1239/2019). Because of the retrospective evaluation of pre-existing medical records, an informed consent was not required. All procedures performed in studies involving human participants were in accordance with the ethical standards of the institutional and/or national research committee and with the 1964 Helsinki declaration and its later amendments or comparable ethical standards.

A consecutive series of 1052 hips in 982 patients with index surgery between 2014 and 2019 was analysed and the medical records until 90 days post-operative were evaluated. Inclusion criteria were defined as end-stage primary osteoarthritis of the hip treated with THA performed via a minimally invasive anterolateral approach using the same type of cementless, curved short stem (Fitmore® stem, ZimmerBiomet, Warsaw, IN, USA), and cementless titanium press-fit cup with or without screws (Allofit®/-S, ZimmerBiomet, Warsaw, IN, USA). Other diagnoses such as avascular necrosis, hip dysplasia, or secondary osteoarthritis were excluded of the study. In total, 160 implantations in 160 patients were excluded due to the pre-operative diagnoses. Fourteen implantations in 14 patients were lost to follow-up. Therefore, 878 implantations in 808 patients were included in this study. Patients were categorised by body mass index (BMI) according to the World Health Organization [19]. The group of underweight patients (BMI > 18.5 kg/m^2^) consisted of nine patients and was therefore brought together with normal weight patients (BMI 18.5 to < 25 kg/m^2^) and was formed as group I (BMI < 25 kg/m^2^) and consisted of 242 implantations (27.6%). Group II (BMI 25 to < 30 kg/m^2^; overweight) consisted of 380 implantations (43.3%); group III (BMI 30 to < 35 kg/m^2^; obese) of 169 implantations (19.2%); group IV (BMI ≥ 35–39.99 kg/m^2^; severely obese) of 68 implantations (7.7%); and group V (BMI ≥ 40 kg/m^2^) of 19 implantations (2.2%). Demographics of age, sex, and the American Society Anaesthesiologists (ASA) score are listed in Table 1.

**Table 1:** Patient demographics for each BMI group; bold values signal statistically significant values in testing

Patient characteristics	Group I BMI ≤ 24.99 kg/m^2^	Group II BMI 25 to < 30 kg/m^2^	Group III BMI 30 to < 35 kg/m^2^	Group IV BMI 35 to < 40 kg/m^2^	Group V BMI ≥ 40 kg/m^2^	P value
Number of hips	242 (27.6%)	380 (43.3%)	169 (19.2%)	68 (7.7%)	19 (2.2%)	
Females/males	160/82	181/199	88/81	39/29	11/8	** < 0.001**
Average age (± SD)	68.07 (± 11.33)	66.68 (± 9.94)	66.94 (± 9.31)	67.66 (± 10.54)	65.17 (± 9.17)	0.178
ASA score	61 ASA I 137 ASA II 44 ASA III	82 ASA I 225 ASA II 73 ASA III	12 ASA I 106 ASA II 49 ASA III 2 ASA IV	3 ASA I 39 ASA II 25 ASA III 1 ASA IV	10 ASA II 9 ASA III	** < 0.001**

### Surgical procedure and implants

Surgical procedures were carried out at the author’s institution by surgeons with different levels of experience including 11 consultants and seven residents. All consultants perform more than 50, all senior consultants more than 100 arthroplasties per year. Resident surgeries were done under the guidance of a consultant. In all cases, a minimally invasive anterolateral Watson-Jones approach in supine position on a standard operating table under laminar flow was performed [20]. Extremity preparation was performed with threefold antiseptic scrub with alcohol disinfectant. Draping with sterile adhesive surgical iodine film was used. A skin incision was centred over the greater trochanter. An incision at the border between the Tensor fasciae latae and the Tractus iliotibilias was performed. Then, the Watson-Jones interval between Tensor fasciae latae and Gluteus medius was bluntly dissected. A capsulectomy was performed in each case. The standardised peri- and postoperative protocol was identical in all cases, including single-shot antibiotics (Cefuroxime 1.5 g i.v. directly pre-operatively), weight-bearing as tolerated from the first postoperative day on, Indomethacin 75 mg twice daily for the prevention of heterotopic ossification on day one to four post-operatively, and 40 mg low-molecular weight heparin or Rivaroxaban 10 mg for 28 days post-operatively as venous thromboembolic event prophylaxis.

In all patients, a cementless, curved short stem (Fitmore® stem, ZimmerBiomet, Warsaw, IN, USA) was digitally templated using mediCAD® version 5.1 (Hectec GmbH, Altdorf, Germany). Fitmore® hip stem is a titanium alloy stem (Ti Al6V4) that has a porolock Ti-VPS coating in the proximal part to enhance bone ingrowth and is available in four different neck angle options (127°, 129°, 137°, 140°) [21]. A cementless titanium press-fit cup with or without screws (Allofit®/-S, ZimmerBiomet, Warsaw, IN, USA) was used in all patients.

### Complications and outcome parameters

All electronically saved and archived medical records were reviewed including operative reports, post-operative notes, discharge summaries, and post-operative medical records. Data included age, weight, height, BMI, sex, operated side, length of operation, length of stay, readmission, re-operation, revision, allogenic transfusion, American Society Anaesthesiologists (ASA) Score, and discharge to home or to a rehabilitation facility. All complications within a 90-day post-operative period were collected. According to the hospital’s standard procedure, transfusion criteria were either a post-operative haemoglobin (Hb) level lower than 7 or 8 g/dl, with the patient being hemodynamically symptomatic. Haemoglobin and haematocrit were measured 24 hours, 48 hours, and five days post-operatively. Blood loss was calculated as described by Charrois et al. [22].

Complications were recorded as surgery-related complications or medical complications. Surgical complication was defined as fracture, periprosthetic joint infection (PJI), nerve lesion (confirmed by electroneurography), wound dehiscence, and haematoma that lead to an intervention such as puncture or surgical intervention. Re-operation was defined as operation without change of prosthesis components. Revision was defined as change of modular parts or removal of the components. Readmission was defined as an additional unplanned in-patient treatment related to index surgery. Medical complications were classified according to the adapted DINDO-Classification by Sink et al. [23]. Medical complications were recorded and classified according to the grades I–V according to severity of complication. All surgical and medical complications were grouped into adverse events and serious adverse events. Adverse events were defined as any unexpected medical occurrence in a patient, which does not necessarily have a causal relationship with the treatment. Serious adverse events were defined as any unexpected medical occurrence (at any dose) in the operative period, which resulted in death, went life-threatening, required inpatient hospitalisation, or prolongation of existing hospitalisation, or resulted in persistent or significant disability/incapacity.

### Statistics

Descriptive statistical analysis was conducted for age, gender, indication, ASA score, and BMI groups by providing absolute/relative frequencies for nominal and ordinal variables and additionally presenting summary statistics as well as measures for variability for metric variables. Kruskal–Wallis test was performed for ASA score, DINDO classification, average age at operation, average hospital stays, average length of stay, and average blood loss stratified by BMI. Post hoc calculations with Bonferroni correction were carried out in case of significant differences. In order to test gender, allogenic transfusion, extended administration, discharge at home, all surgical and medical complications, reoperation, revision, and readmission a Fisher’s exact test were carried out. Post hoc calculation with Bonferroni correction was calculated in case of a significant difference. Binary logistic regression models for dichotomous outcomes were estimated to model the effect of obesity on peri-operative complications, while controlling for age, sex, and ASA scores and surgeon’s experience. The odds ratio and the corresponding 95% confidence interval are reported for every explanatory variable. In the category wound dehiscence, haematoma, and re-operation, BMI group III was used as reference group because none of these two complications occurred in patients with a BMI < 25 kg/m^2^. Surgery performed by residents were included in order to achieve a higher number of participants. In order to evaluate a negative effect of lacking experience on surgical complications, logistic regression was performed to evaluate surgical experience. Performing surgeons were assigned to two groups (resident; consultant) according to their education level. Statistical analysis was calculated with SPSS version 26 (IBM SPSS statistics, Chicago, IL, USA). A p value < 0.05 was considered as statistically significant.

## Results

The patient demographics are shown in Table 1. Statistical analysis for gender difference showed a significant female predominance (p < 0.001). Mean age was comparable in all BMI groups (p = 0.178). ASA score was significantly different (p < 0.001).

Average length of hospital stays and discharge at home showed no statistical significance for BMI (p = 0.150; p = 0.266). Average length of operation was significantly different between BMI groups (p < 0.001). Average operation time increased significantly in obese, severely, and morbidly obese patients (p < 0.001). Post hoc calculations for average operation time showed significant differences after Bonferroni correction between BMI group I and III, I and IV, I and V, and for group II and IV. Average blood loss also showed significant differences between the different BMI groups (p < 0.001). Post hoc calculations for average blood loss showed significant differences after correction between BMI group I and III, I and IV, I and V, II and IV, II and IV, and for group III and V. Testing for differences in allogenic blood transfusion showed statistical significance (p = 0.027) for group I after Bonferroni correction. However, post hoc calculation with Bonferroni correction showed no significant difference for allogenic transfusion between the BMI groups. Testing for medical complication according to DINDO classification showed a statistical significance for group IV (p = 0.035). Full results for average hospital stay, discharge at home, average length of operation, and blood loss and allogenic blood transfusion are shown in Table 2.

**Table 2:** Results of discharge at home, average hospital stays, length of operation and blood loss, transfusion rate, and DINDO classification; absolute frequencies are provided for nominal and ordinal variables. Mean value as well as standard deviation (SD) is provided for metric variables. Bold values indicate significant results in post hoc testing with Bonferroni correction

Hospital data	Group I BMI ≤ 24.99 kg/m^2^	Group II BMI 25 to < 30 kg/m^2^	Group III BMI 30 to < 35 kg/m^2^	Group IV BMI 35 to < 40 kg/m^2^	Group V BMI ≥ 40 kg/m^2^	P value
Home/SNF	176/66	300/80	130/39	47/21	15/4	0.266
Average length of stay; d (SD)	7.82 (± 2.18)	8.03 (± 2.47)	8.36 (± 3.29)	9.82 (± 6.6)	15.21 (± 28.04)	0.150
Average length of operation, min (± SD)	64.16 (± 28.3)	70.02 (± 23.97)	**73.35 (± 24.27)**	**80.39 (± 24.42)**	**85.36 (± 25.92)**	** < 0.001**
Average blood loss, ml (± SD)	271.98 (± 109.22)	319.95 (± 124.54)	**351.69 (± 144.88)**	**408.79 (± 198.98)**	**504.21 (± 209.9)**	** < 0.001**
Number of patients receiving allogenic transfusion	**19 (7.9%)**	13 (3.4%)	4 (2.4%)	2 (2.9%)	2 (10.5%)	**0.027**
DINDO classification	DINDO I 2 DINDO II 6	DINDO II 11	DINDO II 4 DINDO IV 1 DINDO V 1	**DINDO II 7**	DINDO II 2	**0.035**

Test results for the different complications are provided in Table 3. Statistically significant test results are given for general surgical complication (p = 0.015), deep infection (0.001), and revision for any reason (0.013). A statistical significance was found for wound dehiscence as well (p = 0.001). Adverse events were significantly higher in patients in severely obese patients (p = 0.005).

**Table 3:** List of complications within 90 days from index surgery; *BMI*, body mass index, kg/m^2^

Complications	Total	Group I BMI ≤ 24.99 kg/m^2^	Group II BMI 25 to < 30 kg/m^2^	Group III BMI 30 to < 35 kg/m^2^	Group IV BMI 35 to < 40 kg/m^2^	Group V BMI ≥ 40 kg/m^2^	P value
Surgical complication	57 (6.5%)	10 (4.1%)	22 (5.8%)	10 (7.1%)	**17 (14.7%)**	3 (15.8%)	**0.015**
Medical complication	34 (3.9%)	8 (3.3%)	11 (2.69%)	6 (3.6%)	**7 (10.3%)**	2 (10.5%)	**0.035**
Femoral nerve lesion	3 (0.3%)	1 (0.4%)	1 (0.3%)	1 (0.6%)	0 (0.0%)	0 (0.0%)	0.845
Fractures	10 (1.1%)	2 (0.2%)	6 (0.7%)	2 (1.2%)	0 (0.0%)	0 (0.0%)	0.870
Deep infection (PJI)	12 (1.4%)	1 (0.4%)	4 (1.1%)	1 (0.6%)	**4 (5.9%)**	**2 (10.5%)**	**0.001**
Hematoma	7 (0.8%)	0 (0.0%)	5 (1.3%)	1 (0.5%)	1 (1.5%)	0 (0.0%)	0.318
Dislocations	9 (1.0%)	6 (2.5%)	2 (0.5%)	0 (0.0%)	1 (1.5%)	0 (0.0%)	0.088
Wound dehiscence	17 (1.9%)	0 (0.0%)	5 (1.3%)	7 (4.1%)	4 (5.9%)	**1 (5.3%)**	**0.001**
Reoperation	4 (0.5%)	0 (0.0%)	2 (0.5%)	0 (0.0%)	2 (2.9%)	0 (0.0%)	0.071
Revision	17 (1.9%)	2 (0.8%)	6 (1.6%)	3 (1.8%)	4 (5.9%)	2 (10.5%)	**0.013**
Readmission	22 (2.5%)	7 (2.9%)	7 (1.8%)	3 (1.8%)	4 (5.9%)	1 (5.3%)	0.201
Adverse events	49 (5.6%)	7 (2.9%)	19 (5.0%)	11 (6.5%)	**9 (13.2%)**	3 (6.1%)	**0.005**
Serious adverse events	42 (4.8%)	10 (4.1%)	15 (3.9%)	8 (19.0%)	7 (16.7%)	2 (10.5%)	0.133

Bold values signal statistically significant values in testing

Table 4 presents the results for logistic regression analyses for dichotomous complication outcomes as dependent variables. Surgery performed by a surgeon in training led to a significantly increased risk of allogenic transfusion (OR = 2.683, CI 1.387–5.192). Male sex was significantly lower at risk for overall surgical complication (OR = 0.489, CI 0.268–0.891). A BMI between 25 and 35 kg/m^2^ was associated with a lower risk rate of allogenic blood transfusion (OR = 0.430, CI 0.205–0.902; OR = 0.273, CI 0.089–0.840). Patients with BMI > 35 kg/m^2^ showed a significantly higher risk for general surgical complication (OR = 4.365, CI 1.674–11.382; OR = 4.985; CI 1.188–20.921), deep infection (OR = 21.687, CI 2.203–213.520; OR = 57.653; CI 4.132–804.525), and revision (OR = 8.793, CI 1.489–51.924; OR = 20.708; CI 2.430–176.446). Higher ASA score was detected as a risk factor for medical complications according to DINDO classification (OR = 2.531, CI 1.333–4.806) and re-operation (OR = 10.476, CI 1.245–88.165). Age was detected as a risk factor for adverse (OR = 3.490, CI 1.264–9.638) and serious adverse events (OR = 1.094, CI 1.009–1.187). Severely obese (OR = 4.345, CI 1.506–12.533) and morbidly obese (OR = 5.365, CI 1.190–24.099) patients showed a higher risk for adverse events as well.

**Table 4:** Results of logistic regression analysis using several dependent variables are listed per column. The estimated odds ratio as well as the corresponding confidence interval is listed per explanatory variable row-wise with additional information on the p value using a) P < 0.05, b) P < 0.01; *NV*, no variation (none of these patients in this category experienced these complications

Dependent variables	Allogenic transfusion	Surgical complication	Fractures	Deep infection	Haematoma	Dislocation	Wound dehiscence	Readmission	Re-operation	Revision	Serious adverse events
Age	1.012 0.976–1.049	1.013 0.981–1.045	1.039 0.960–1.125	1.082 1.001–1.169	1.026 0.931–1.131	0.969 0.906–1.037	0.985 0.932–1.041	0.978 0.934–1.023	0.922 0.809–1.050	1.061 0.997–1.129	1.094 1.009–1.187_a)_
Sex	0.515 0.248–1.071	0.489^a)^ 0.268–0.891	0.298 0.061–1.441	1.151 0.337–3.931	1.632 0.343–7.768	0.392 0.076–2.015	0.288 0.090–1.041	0.540 0.212–1.377	1.142 0.123–10.617	0.785 0.276–2.229	0.571 0.286–1.141
ASA	1.178 0.664–2.091	0.953 0.582–1.560	1.583 0.507–4.940	0.553 0.184–1.660	3.461 0.870–13.764	0.755 0.234–2.436	0.667 0.263–1.691	1.159 0.542–2.478	10.476^a)^ 1.245–88.165	0.842 0.345–2.053	1.112 0.631–1.959
Surgeon’s experience	2.683^b)^ 1.387–5.192	1.119 0.601–2.081	1.284 0.325–5.070	2.675 0.801–8.930	1.313 0.245–7.047	NV	0.834 0.263–2.644	0.943 0.341–2.607	3.629 0.451–29.215	1.834 0.656–5.126	1.447 0.731–2.863
BMI < 25	Reference category	Reference category	Reference category	Reference category	NV	Reference category	NV	Reference category	NV	Reference category	Reference category
BMI (25 to 29.99)	0.430^a)^ 0.205–0.902	1.606 0.740–3.486	2.239 0.440–11.386	2.617 0.285–24.061	Reference category	0.265 0.052–1.364	Reference category	0.708 0.242–2.074	Reference category	2.031 0.400–10.309	1.056 0.458–2.434
BMI (30 to 34.99)	0.273^a)^ 0.089–0.840	1.999 0.822–4.859	1.527 0.202–11.547	1.949 0.116–32.894	0.352 0.038–3.225	NV	3.426 1.023–11.476	0.619 0.153–2.502	NV	2.616 0.415–16.513	1.281 0.474–3.460
BMI (35 to 39.99)	0.324 0.071–1.477	4.365^b)^ 1.674–11.382	NV	21.687^b)^ 2.203–213.520	0.858 0.089–8.260	0.856 0.090–8.118	5.186a) 1.258–21.376	2.086 0.561–7.761	2.969 0.343–25.675	8.793a) 1.489–51.924	2.831 0.984–8.150
BMI ≥ 40	1.400 0.280–6.989	4.985a) 1.188–20.921	NV	57.653b) 4.132–804.525	NV	NV	4.213 0.423–41.967	1.695 0.187–15.366	NV	20.708a) 2.430–176.446	3.179 0.605–16.718

## Discussion

Obesity was identified as a major risk factor for peri- and post-operative complications in arthroplasty [24–28]. However, the knowledge about obesity as a risk factor in minimally invasive approaches is limited and concentrates predominantly on DAA. Purcell et al. [29] found a significantly increased risk for PJI in DAA in severely obese patients. In a matched-pair-study, Antoniadis et al. [30] found a higher number of surgical complications in DAA in severely obese patients but comparable to other approaches. Our findings are comparable regarding general surgical complication with 6.5% compared to 5.6% in THA via DAA [24].

Total blood loss was significantly higher in overweight, obese, severely, and morbidly obese patients. However, this did not affect the transfusion rate in these groups The rate of allogenic transfusion was significantly lower in patients with BMI ≥ 25 kg/m^2^ comparable to DAA [24]. One risk factor for the need of allogenic transfusion was surgeon’s experience. Surgery carried out by residents increased the risk for postoperative transfusion significantly. This study also showed a higher risk for allogenic transfusion with higher ASA scores.

The rate of deep infections was significantly higher in severely and morbidly obese patients. Statistical analysis showed a clear cut-off with a BMI ≥ 35 kg/m^2^ with the highest risk for morbidly obese patients. The risk for PJI according to BMI differs in the literature. Friedman et al. and Shohat et al. [31] report a BMI threshold of ≥ 40 kg/m^2^ for PJI in total joint arthroplasty (TJA) within the first 90 days. Hartford et al. [24] reported a higher risk for PJI in THA using the DAA in morbidly obese patients as well. However, obese and severely obese patients formed one group (BMI ≥ 30–39.99 kg/m^2^). We report results and an odds ratio for severely and morbidly obese patients, postulating that a BMI ≥ 35 kg/m^2^ is clearly the threshold for PJI in anterolateral MIS THA.

Another major finding in the presented study is increased operation time with increasing BMI. Operation time was significantly higher in patients with a BMI > 25 kg/m^2^ and highest in severely obese patients. The increased operation time in MIS anterolateral approach is comparable to DAA [24]. Increased operation time is a known risk factor for PJI in TJA [32–34]. Each 20-minute increase in operative time is associated with nearly a 25% increased risk of subsequent PJI in primary TJA [32]. This study supports these findings with a statistically significant higher risk for PJI in severely and morbidly obese patients combined with a statistically significant increased operation time in severely obese patients.

Obesity is also seen to be a risk factor for wound dehiscence [24, 35]. We report wound dehiscence as a complication only in patients with a BMI > 25 kg/m^2^ with the highest rate in severely obese patients. Logistic regression also shows a clear risk for wound complication especially for severely obese patients. Hartford et al. [24] report a rate of wound dehiscence in DAA in the general cohort with 0.7% and 8.3% for morbidly obese patients. Wenz et al. [36] reported a 2% wound complication rate with a mini-incision anterolateral approach. In a meta-analysis, Aggarwal et al. [10] found a reported rate of wound complication of 2.7% in posterior approach and 5.7% with DAA. Our study reports a rate of wound complications of 1.9% for a MIS anterolateral approach comparable to other results for the same type of approach [36]. Our findings are also comparable to posterior approach [10].

MIS anterolateral short stem THA showed a dislocation rate of 1.1%. This rate is comparable to 0.6% for DAA [24]. BMI was not identified as a risk for dislocations in MIS anterolateral approach. The general influence of obesity on dislocation is not fully clarified. While obesity is not seen to lead to a higher risk for dislocation [24, 30], other studies report a higher rate of dislocations for obese patients [26, 37]. We postulate that the use of minimally invasive approaches reduces the risk of dislocation independently of the BMI.

Iwata et al. [17] found an increased risk of greater trochanteric fracture in supine anterolateral approach in obese patients. Herndon et al. [16] reported a rate of periprosthetic fractures with 8.7% in anterolateral approach. Our results do not support these findings with a generally low number of periprosthetic fractures without statistically significant results in testing and logistic regression.

The risk for revision surgery was significantly higher in severely and morbidly obese patients. This is likely to be a result of the higher rate of PJI in patients with a BMI ≥ 35 kg/m^2^. A higher rate of PJI automatically results in a higher revision rate of one-stage or two-stage revisions. BMI ≥ 35 kg/m^2^ is a clear risk factor for PJI and revision in MIS anterolateral approach. However, a higher rate of revisions did not lead to higher readmission rate. For readmission, no significant differences between the BMI groups were found. One factor for these findings could be the average hospital stay, that is, generally significantly longer compared to other studies [24]. Therefore, a significant number of patients underwent revision surgery within the first hospital stay.

Large studies show an increased risk for peri-operative complications such as PJI, dislocation, wound dehiscence, or fractures with increasing BMI [17, 24, 25, 38]. Increased complications above a BMI of 40 kg/m^2^ are reported by several studies independent from the used surgical approach [24, 25, 38, 39]. The posterior approach is associated with less peri-operative complications compared to the direct lateral approach in morbidly obese patients [40]. Severely and morbidly obese patients are associated with medical comorbidities such as diabetes mellitus and cardiovascular disorders. Moreover, THA in obese patients may be more surgically demanding as the voluminous deep adipose tissue, weak fatty-infiltrated peri-articular soft-tissue envelope impairs visibility and surgical exposure. Especially the risk for PJI is associated in severely or morbidly obese patients independently from the used approach. Additionally, MIS approaches show low rates of dislocations and allow early mobilisation. Therefore, we conclude that MIS anterolateral approach is feasible in severely and morbidly obese patients.

Limitations of this study is the retrospective study design. Due to the retrospective study design, we cannot give adequate results for the rate of patients with diabetes, active smokers, or other medical conditions influencing risk for complications. However, the logistic regression included multiple variables apart from BMI in order to evaluate additional risk factors. ASA score was available in every patient, which is a commonly used score for evaluating the general condition of a patient undergoing surgery. Another limitation is the very short follow-up of only 90 days. However, the aim of this study was to focus on early postoperative complications. Therefore, the short follow-up period was deliberately chosen. Additionally, the results are only applicable in short stem THA via a MIS anterolateral approach in supine position. Further research is needed to give clear evidence, if anterior-based approaches lead to a higher number of PJI in severely and morbidly obese patients or if the increased risk is independent from the approach.

## Conclusion

The risk for early PJI and overall surgical complications in MIS anterolateral approach is significantly increased in severely and morbidly obese patients. This leads to a significantly higher risk for revision surgery after index surgery within the first 90 days. A BMI above 35 kg/m^2^ is the clear threshold for increased risk of PJI in MIS anterolateral THA with a short curved stem. As the surgical complications are comparable to other approaches, MIS anterolateral short stem THA is also feasible with increasing BMI.

## Authors Contribution

M. Luger: wrote the manuscript, performed the statistical analysis, designed the study, acquisition of data, interpretation of the data.

R. Hochgatterer: revised the manuscript.

L. Pisecky: jointly conceived the study, interpretation of the data.

C. Schopper: involved in the acquisition of data, interpretation of the data.

J. Allerstorfer: involved in the acquisition of data, interpretation of the data.

A. Klasan: jointly conceived the study, performed statistical analysis, edited the manuscript.

T. Gotterbarm: revised the manuscript.

B. Schauer: jointly conceived the study, interpretation of data, edited the manuscript.

## Data Availability

Data and materials are available on request.
